# Effect of Gold Nanoparticle Size on Regulated Catalytic Activity of Temperature-Responsive Polymer−Gold Nanoparticle Hybrid Microgels

**DOI:** 10.3390/gels10060357

**Published:** 2024-05-22

**Authors:** Palida Pongsanon, Akifumi Kawamura, Hideya Kawasaki, Takashi Miyata

**Affiliations:** 1 Department of Chemistry and Materials Engineering, Kansai University, Suita, Osaka 564-8680, Japan; k869374@kansai-u.ac.jp (P.P.); akifumi@kansai-u.ac.jp (A.K.); hkawa@kansai-u.ac.jp (H.K.); 2 Organization for Research and Development of Innovative Science and Technology, Kansai University, Suita, Osaka 564-8680, Japan

**Keywords:** temperature-responsive polymer, gold nanoparticle, organic–inorganic hybrid, catalytic activity, lower critical solution temperature

## Abstract

Gold nanoparticles (AuNPs) possess attractive electronic, optical, and catalytic properties, enabling many potential applications. Poly(*N*-isopropyl acrylamide) (PNIPAAm) is a temperature-responsive polymer that changes its hydrophilicity upon a slight temperature change, and combining PNIPAAm with AuNPs allows us to modulate the properties of AuNPs by temperature. In a previous study, we proposed a simpler method for designing PNIPAAm–AuNP hybrid microgels, which used an AuNP monomer with polymerizable groups. The size of AuNPs is the most important factor influencing their catalytic performance, and numerous studies have emphasized the importance of controlling the size of AuNPs by adjusting their stabilizer concentration. This paper focuses on the effect of AuNP size on the catalytic activity of PNIPAAm–AuNP hybrid microgels prepared via the copolymerization of *N*-isopropyl acrylamide and AuNP monomers with different AuNP sizes. To quantitatively evaluate the catalytic activity of the hybrid microgels, we monitored the reduction of 4-nitrophenol to 4-aminophenol using the hybrid microgels with various AuNP sizes. While the hybrid microgels with an AuNP size of 13.0 nm exhibited the highest reaction rate and the apparent reaction rate constant (*k*_app_) of 24.2 × 10^−3^ s^−1^, those of 35.9 nm exhibited a small *k*_app_ of 1.3 × 10^−3^ s^−1^. Thus, the catalytic activity of the PNIPAAm–AuNP hybrid microgel was strongly influenced by the AuNP size. The hybrid microgels with various AuNP sizes enabled the reversibly temperature-responsive on–off regulation of the reduction reaction.

## 1. Introduction

Over the past decade, metal nanoparticles have been the subject of extensive research owing to their versatile properties as innovative materials. In particular, gold nanoparticles (AuNPs) possess attractive properties, such as electronic, optical, and catalytic properties [1,2,3], which are suitable for use in the medical [4] and environmental fields [5]. Various applications of AuNPs have been explored, including sensors [6,7] and bioimaging [8,9]. Additionally, the use of AuNPs as catalysts has been studied for hydration [10,11], coupling [12,13], oxidation [14,15,16], and reduction reactions [17,18,19]. Recently, the combination of nanoparticles with biomolecules [20], other noble metals [21,22], and responsive polymers [23,24,25,26,27] has also attracted much attention for stabilizing AuNPs in aqueous media and regulating their properties. For example, the hybridization of AuNPs with various functional polymers with thiol groups enhances the stability of AuNPs in aqueous media and provides new features, especially responsive properties, enabling the regulation of their unique properties [25,26,28,29,30,31,32,33].

Responsive polymers have attractive functions, such as natural feedback systems, capable of drastic changes in their structures and properties, such as conformation and hydrophilicity, in response to stimuli. Various responsive polymers, gels, and particles have been strategically designed owing to their versatile applications and unique abilities to respond to various external stimuli [34,35], such as temperature [26,30,36,37], biomolecules [38,39,40], and multiple stimuli such as ions, pH, light, and temperature [41,42,43,44]. Combining AuNPs with stimuli-responsive polymers allows us to control their properties, stability in aqueous media, and recovery using stimuli, such as temperature and light. For example, AuNPs were immobilized within stimuli-responsive hydrogels for the development of sensors and catalysis, which can regulate their properties in response to external stimuli [7,31,45,46,47,48,49]. Such stimuli-responsive hybrids, composed of stimuli-responsive polymers and AuNPs, have attracted significant attention owing to their unique properties and many potential applications. In the preparation of stimuli-responsive polymer–AuNP hybrids, PNIPAAm has emerged as a prominent temperature-responsive polymer as it has a lower critical solution temperature (LCST) of 34 °C. In general, PNIPAAm–AuNP hybrids were prepared using the following three methods: First, the “grafting-to” method involves the introduction of thiol-terminated PNIPAAm onto the surface of AuNPs. Second, the “grafting-from” method involves the polymerization of NIPAAm monomers from the initiator-introduced surfaces of AuNPs. Both methods enable the production of temperature-responsive core-shell nanoparticles with an AuNP core and PNIPAAm chain shell [45,50,51]. Third, PNIPAAm–AuNP hybrids can be prepared by the reduction in Au ions within PNIPAAm microgels and hydrogels [25,26,52,53,54]. In general, while PNIPAAm covalently binds to the AuNP surfaces in the PNIPAAm–AuNP hybrids prepared using the first and second methods, AuNPs are physically entrapped within the PNIPAAm networks in the hybrids prepared using the third method. Unlike their standard method, our previous study proposed a simple method to prepare PNIPAAm–AuNP hybrids in which AuNPs are covalently linked with microsized PNIPAAm networks [55]. This method uses an AuNP monomer with polymerizable groups. We prepared AuNP monomers by attaching hydrophobic polymerizable groups and hydrophilic carboxyl groups onto the AuNP surface. The PNIPAAm–AuNP hybrid microgels were prepared by the copolymerization of the AuNP monomer with NIPAAm. The reduction of 4-nitrophenol (4-NP) to 4-aminophenol (4-AP) was catalyzed by the hybrid microgels at 25 °C but not at 40 °C.

Various factors such as size, shape, composition, the coordination number, and the surface chemistry of AuNPs play a crucial role in determining their catalytic activity [26,56,57,58,59,60,61,62,63,64]. As the size of AuNPs is the most important factor influencing their catalytic performance among these factors, significant efforts have been made to optimize the size and enhance the catalytic activity. Numerous studies have emphasized the importance of controlling the size of AuNPs by adjusting the stabilizer concentration [59,63,65,66]. The size tunability of AuNPs is important because it directly affects their catalytic activity. In general, AuNPs with a smaller size exhibit high catalytic activity owing to their larger specific surface area than those with a larger size. This is attributed to the fact that the increased surface area allows for a more efficient adsorption of reactant molecules onto the AuNP surface. In addition, AuNPs with a smaller size have more active sites because their reduced dimensions correspond to lower coordination numbers. Furthermore, researchers have recognized the critical role of the optimized size of AuNPs, particularly when they are loaded onto supporting materials. Such integration aims to enhance catalytic activity and selectivity through charge transfer between the AuNPs and the support. As highlighted by Cao et al., the size and shape of AuNPs depend on their catalytic performance in oxidation and reduction reactions [67]. Lin et al. reported the highest performance of Au/Al_2_O_3_ with a diameter of 3.4 nm among those of various sizes in the reduction reaction of 4-NP [60]. Similarly, Valden et al. and Laoufi et al. observed enhanced catalytic activity in CO oxidation with 2–3 nm AuNPs on TiO_2_ [68,69]. Bokhimi et al. also reported that the activity of AuNPs on rutile increased with decreasing the size from 6 to 3 nm [70]. In addition, Liang et al. highlighted the nuanced relationship between AuNP size, crystallinity, and defect concentration [62]. In contrast, Fenger et al. reported a unique relationship between AuNP size and catalytic activity for the reduction in 4-NP, in which the catalytic activity of cetyltrimethylammonium bromide-stabilized AuNPs increased with an increase in size from 3 to 13 nm, but decreased with an increase in size from 13 to 56 nm [59]. This might indicate that AuNPs with extremely small sizes are unsuitable catalysts for certain reactions. This is supported by the results reported by Donoeva et al. [58], in which triphenylphosphine-stabilized AuNPs with a size of only >2 nm on SiO_2_ exhibited catalytic activity in the oxidation of cyclohexene. Thus, many previous studies have indicated that the optimization of AuNP size is important for preparing stimuli-responsive polymer–AuNP hybrids with high catalytic activity.

In this study, we prepared PNIPAAm–AuNP hybrid microgels using AuNP monomers with various AuNP sizes, which is an important factor that determines their catalytic activity in the reduction of 4-NP to 4-AP. AuNP monomers with polymerizable groups were prepared by the attachment of both acryloyl and carboxy groups onto the surface of AuNPs with various diameters. This paper focuses on the effect of AuNP size on the temperature-responsive catalytic activity of hybrid microgels prepared by the copolymerization of NIPAAm and AuNP monomers. The temperature-responsive catalytic activity of the hybrid microgels with different AuNP sizes was investigated by monitoring the reduction reaction of 4-NP upon cycling at different temperatures. An important advance of this study is that the reversibly temperature-responsive catalytic activity of the hybrid microgels can be enhanced using AuNP monomers with small AuNP sizes.

## 2. Results and Discussion

### 2.1. Preparation of AuNP Monomers with Various Sizes

**Figure 1:**
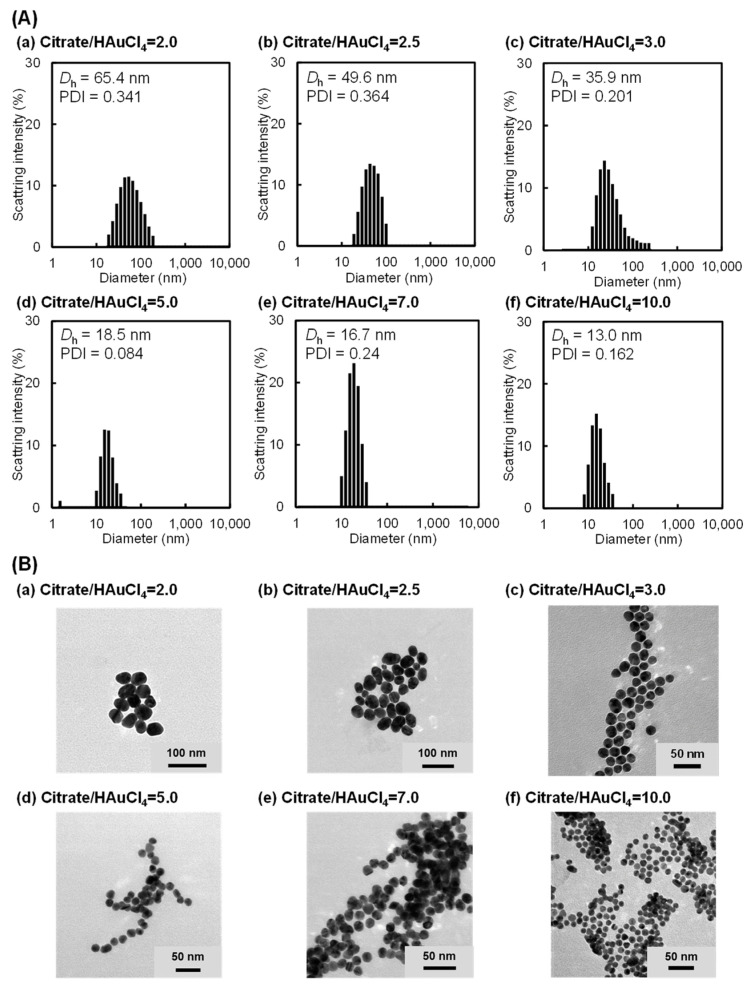
(**A**) Size distribution and (**B**) TEM images of citrate–AuNPs prepared with various citrate/HAuCl_4_ ratios. The hydrodynamic size (*D*_h_) and size distribution of the citrate–AuNPs were determined by DLS measurements. The citrate–AuNP concentration in the aqueous dispersion was 0.35 mg/mL.

The molar ratio of sodium citrate hydrate to HAuCl_4_ (citrate/HAuCl_4_ ratio) is a crucial factor that strongly influences the formation of AuNPs [71,72,73,74]. In this study, citrate–AuNPs of various sizes were prepared by varying the citrate/HAuCl_4_ ratio (Scheme S1). Figure S1 shows the photographs and UV–Vis spectra of aqueous dispersions containing citrate–AuNPs prepared with various citrate/HAuCl_4_ ratios. An aqueous dispersion of the citrate–AuNPs changed slightly from red to purple upon decreasing the citrate/HAuCl_4_ ratio. Furthermore, the UV–Vis spectra demonstrated that the absorbance peak of the dispersion with citrate–AuNPs prepared at a lower citrate/HAuCl_4_ ratio shifted from 520 to 530 nm, which agrees with the slight change in the color of the dispersions from red to purple. In general, a small shift in the absorbance peak to a longer wavelength indicates the formation of AuNPs with larger sizes [73,74]. These results indicate that the size of citrate–AuNPs increases gradually with a decrease in the citrate/HAuCl_4_ ratio. Furthermore, we measured the size of citrate–AuNPs prepared with various citrate/HAuCl_4_ ratios using DLS measurements and TEM observations. As shown in Figure 1A, DLS measurements revealed that the hydrodynamic sizes (*D*_h_) of citrate–AuNPs prepared at citrate/HAuCl_4_ ratios of 2.0, 2.5, 3.0, 5.0, 7.0, and 10.0 were 65.4, 49.6, 35.9, 18.5, 16.7, and 13.0 nm, respectively. In addition, the TEM images demonstrated that the size of citrate–AuNPs decreased with an increase in the citrate/HAuCl_4_ ratio, as shown in Figure 1B and Figure S2. As a result, we concluded that citrate–AuNPs of various sizes were successfully prepared by tuning the citrate/HAuCl_4_ ratio. The preparation of citrate–AuNPs of various sizes exhibited good reproducibility through the careful control of a few crucial factors, but more careful control of the preparation conditions is required for practical applications and scalability.

**Figure 2:**
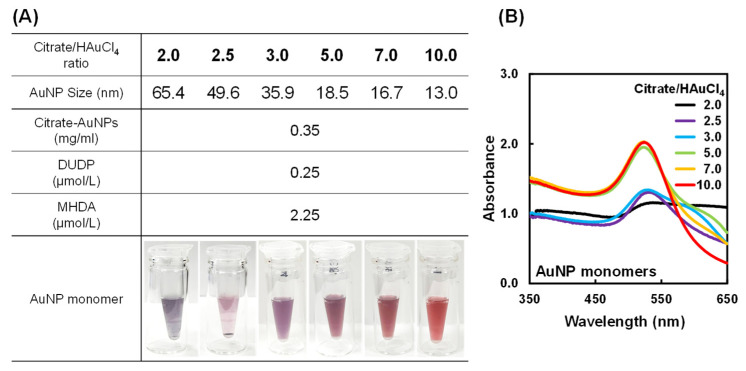
(**A**) Photographs and (**B**) UV–Vis spectra of AuNP monomers designed by attaching DUDP and MHDA to citrate–AuNPs with various citrate/HAuCl_4_ ratios. The concentration of the AuNP monomer in the aqueous dispersion was 0.95 mg/mL.

According to the method reported in our previous paper [55], AuNP monomers were prepared by attaching hydrophobic polymerizable acryloyl and hydrophilic carboxyl groups to the AuNP surface (Scheme S2). First, DUDP with two acryloyl groups was synthesized as a thiol to introduce polymerizable groups onto the surface of AuNPs (Scheme S3). ^1^H-NMR and FT-IR spectroscopy confirmed the successful synthesis of DUDP (Figures S3 and S4). As the introduction of hydrophobic acryloyl groups onto the AuNP surface reduced the stability of the AuNP monomers in water, hydrophilic carboxyl groups were simultaneously introduced by the reaction of MHDA, which contains carboxyl and thiol groups, with the AuNP surface. In a previous study, we found that the optimal molar ratio of DUDP and MHDA was 5 to 45 in preparing AuNP monomers stably dispersed in aqueous media. Therefore, AuNP monomers with various AuNP sizes were prepared with the optimal molar ratio of DUDP and MHDA. Figure 2 shows photographs and UV–Vis spectra of aqueous dispersions of AuNP monomers prepared from citrate–AuNPs of various sizes, which were tuned by the citrate/HAuCl_4_ ratios. The dispersions of AuNP monomers with a citrate/HAuCl_4_ ratio of 2.0 and 2.5 were purple, and their UV–Vis spectra showed a distinct shoulder peak at approximately 600 nm. This indicates that the AuNP monomers with a citrate/HAuCl_4_ ratio of 2.0 and 2.5 aggregated owing to their low stability in aqueous media. In general, AuNPs prepared by the Turkevich method are stably dispersed in aqueous media because of the repulsion of citrate groups on their surfaces. AuNP monomers were prepared through a ligand exchange of the citrate group with DUDP to introduce acryloyl groups to the citrate–AuNP surface. As an acryloyl group is hydrophobic and not charged, its introduction onto the AuNPs through the exchange of the charged citrate group with the hydrophobic acryloyl group lowers the stability of the AuNPs in aqueous media. On the other hand, dispersions of AuNP monomers with a citrate/HAuCl_4_ ratio of more than 2.5 still retained good dispersibility in aqueous media. As citrate–AuNPs with a citrate/HAuCl_4_ ratio of more than 2.5 are stabilized by hydrophilic citrate groups, the resulting AuNP monomers can maintain high stability in aqueous media. Therefore, in this study, AuNP monomers with a citrate/HAuCl_4_ ratio of more than 2.5 were used to prepare PNIPAAm–AuNP hybrids. In this paper, the AuNP monomers from citrate–AuNPs prepared with citrate/HAuCl_4_ ratios of 3.0, 5.0, 7.0, and 10.0, respectively (AuNP size: 35.9, 18.5, 16.7, and 13.0 nm), were named AuNP monomers 1, 2, 3, and 4, respectively. AuNP monomers 1, 2, 3, and 4 were copolymerized with NIPAAm to prepare hybrid microgels with various AuNP sizes.

### 2.2. Preparation of PNIPAAM–AuNP Hybrid Microgels Using AuNP Monomers with Different AuNP Sizes

PNIPAAm–AuNP hybrids were prepared by the copolymerization of AuNP monomers with various AuNP sizes (AuNP monomers 1, 2, 3, and 4) and NIPAAm, according to the method outlined in Scheme S4. AuNP monomers 1, 2, 3, and 4 with AuNP diameters of 35.9, 18.5, 16.7, and 13 nm, respectively, can act as crosslinkers for the formation of hybrids because they have a few acryloyl groups per AuNP. The FT-IR spectrum of the PNIPAAm–AuNP hybrids after purification by centrifugation at 40 °C is similar to that of PNIPAAm (Figure S5). In addition, XPS measurements demonstrated that the C_1s_/Au_4f_ ratio of the PNIPAAm–AuNP hybrids was much greater than that of the citrate–AuNPs (Table S1), indicating the formation of PNIPAAm. Importantly, we detected Au in the PNIPAAm–AuNP hybrids even after the removal of unreacted AuNP monomers by purification. From these results, we concluded that PNIPAAm–AuNP hybrids were successfully prepared using AuNP monomers of various AuNP sizes.

**Figure 3:**
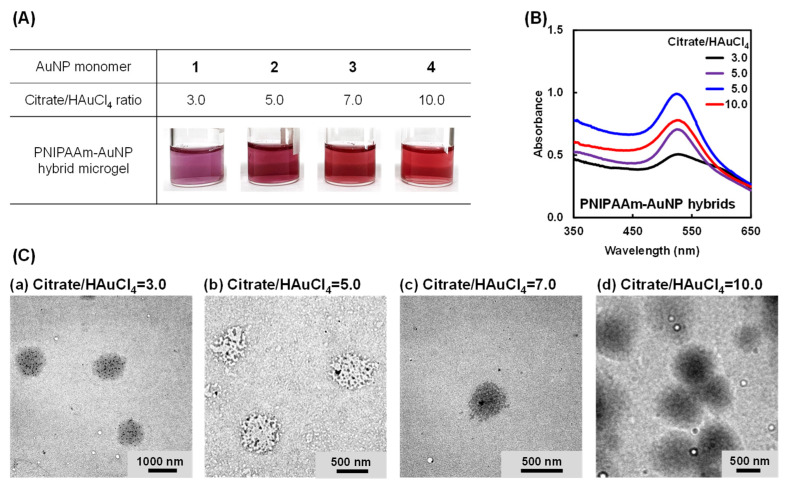
(**A**) Photographs, (**B**) UV spectra, and (**C**) TEM images of PNIPAAm–AuNP hybrid microgels prepared using AuNP monomers 1–4, which were prepared from citrate–AuNPs with citrate/HAuCl_4_ ratios of 3.0, 5.0, 7.0, and 10.0. The concentration of the hybrid microgel in the aqueous dispersion was 31 mg/mL.

Figure 3A,B show photographs and UV–Vis spectra of aqueous dispersions of PNIPAAm–AuNP hybrids prepared using AuNP monomers of various sizes (AuNP monomers 1–4). The photographs demonstrate that the dispersion of the PNIPAAm–AuNP hybrids changed slightly from red to purple with a decreasing citrate/HAuCl_4_ ratio. This might be attributed to the fact that the AuNPs partially aggregated during the copolymerization of the AuNP monomer and NIPAAm. However, no precipitation was observed in any of the dispersions of the hybrids prepared using AuNP monomers 1–4. We investigated the structure of the hybrids by TEM after freeze-drying. The TEM images demonstrate that the hybrids prepared using AuNP monomers 1–4 formed microgels with a diameter of a few hundred nanometers (Figure 3C). Importantly, we observed several AuNPs within the microsized network in the TEM images. DLS measurements demonstrated that the hybrids had a hydrodynamic size (*D*_h_) of a few hundred nanometers and wide size distribution (Figure S6). From Figure S6, we can find no tendency for the size distribution of the hybrids when the citrate/HAuCl_4_ ratio changes. This means that the citrate/HAuCl_4_ ratio is an important factor that determines the size of the citrate–AuNPs, but has no influence on the size of the PNIPAAm–AuNP hybrids. Furthermore, in the TEM image, AuNPs were observed within the microsized PNIPAAm networks after purification, but were not observed outside the networks. This indicates that the AuNPs were covalently immobilized within the microsized networks. As a result, we concluded that the PNIPAAm–AuNP hybrids were microgels in which AuNPs were covalently immobilized within microsized networks of a few hundred nanometers. In addition, the formation of the hybrid microgels without using a chemical crosslinker, such as N,N′-methylene bisacrylamide, means that the AuNP monomers acted as crosslinkers in the copolymerization. Even though N,N′-methylene bisacrylamide is not used, a few acryloyl groups on the AuNP monomer can be copolymerized with the acrylamide group of NIPAAm, followed by the formation of the hybrid microgel.

**Figure 4:**
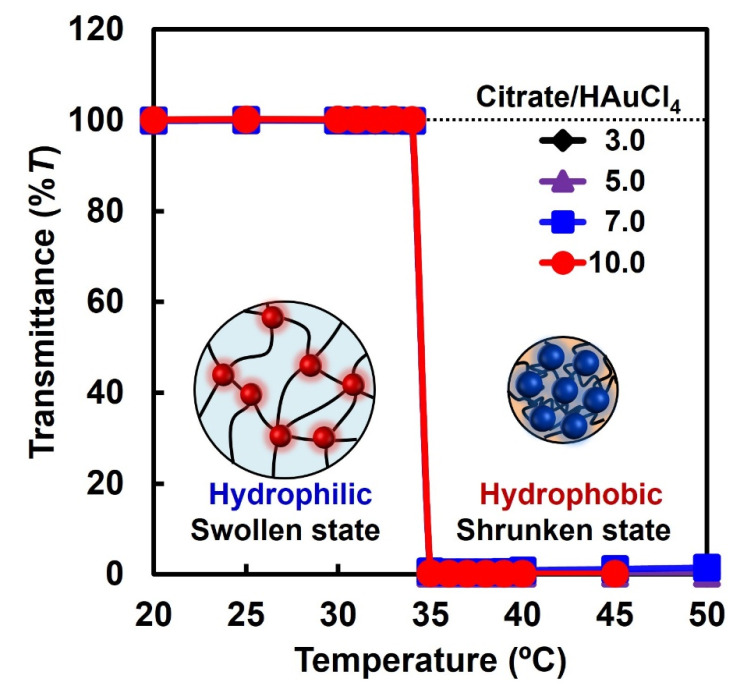
Effect of temperature on the transmittance of dispersions of PNIPAAm–AuNP hybrid microgels with a concentration of 1.0 mg/mL.

PNIPAAm is a typical temperature-responsive polymer, with an LCST of approximately 34 °C. In this study, we prepared PNIPAAm–AuNP hybrid microgels with different AuNP sizes to investigate the effect of the AuNP size on their catalytic activity. Therefore, the effect of temperature on the transmittance of the dispersion of the hybrid microgels, which were prepared using AuNP monomers 1, 2, 3, and 4 with AuNP diameters of 35.9, 18.5, 16.7, and 13 nm, respectively, was investigated to evaluate their temperature responsiveness (Figure 4). At temperatures lower than 34 °C, the aqueous dispersions of the hybrid microgels with a 1.0 mg/mL concentration were almost colorless and transparent (Figure S7A). In contrast, the dispersion was cloudy at temperatures higher than 34 °C (Figure S7B). The sharp decrease in transmittance at 34 °C implies that the hybrid microgel changed from hydrophilic to hydrophobic at 34 °C with increasing temperature, similar to PNIPAAm. Although the PNIPAAm chains of the hybrid microgels expanded in water below 34 °C, they shrank above 34 °C. Importantly, the cloud point was independent of AuNP size in the hybrid microgel. In general, the amount of crosslinker in temperature-responsive gels influences the magnitude of the changes in volume and size, such as the swelling and shrinkage of the gel, but hardly affects the transition temperature. This is because their temperature-responsive behavior is induced by a drastic change in the hydrophilicity/hydrophobicity of the polymer chains and is not influenced by the small amount of the crosslinker. As a result, hybrid microgels with different AuNP sizes had the same cloud point at 34 °C. In the next section, we discuss the effect of AuNP size on the temperature-responsive catalytic activity of the hybrid microgels.

### 2.3. Catalytic Activity of PNIPAAm–AuNP Hybrid Microgels with Various AuNP Sizes

In a previous paper, we proposed a new method to prepare temperature-responsive polymer–AuNP hybrids using an AuNP monomer with polymerizable groups [55]. The resulting PNIPAAm–AuNP hybrid microgels switched their catalytic activity upon changing their temperature. In this study, we designed various AuNP monomers with different AuNP sizes, which is an important factor governing the catalytic activity of AuNPs, and prepared PNIPAAm–AuNP hybrid microgels using their AuNP monomers. This paper focuses on the effect of AuNP size on the temperature-responsive catalytic activity of the hybrid microgels. In general, the reduction of 4-NP to 4-AP is monitored to evaluate the catalytic activity of metal nanoparticles [17,36,42,75]. The reduction reaction of 4-NP can be easily monitored by measuring the absorbance of the sample solution at 400 nm, spectroscopically assigned to 4-NP [17,76]. When the reduction of 4-NP to 4-AP progresses, the absorbance of a sample solution at 400 nm decreases, and that at 300 nm increases simultaneously, corresponding to a color change of the solution from pale yellow to colorless. In this study, we investigated the catalytic activity of the hybrid microgels with different AuNP sizes in the reduction in 4-NP with excess NaBH_4_ by monitoring the absorbance of an aqueous 4-NP solution at 400 nm. Although the aqueous 4-NP solution changed from pale yellow to colorless after the addition of the hybrid microgel at 25 °C, it remained pale yellow at 40 °C (Figure S8). This indicates that the catalytic activity of the hybrid microgels was strongly influenced by temperature. To monitor the reduction of 4-NP to 4-AP using the hybrid microgels, we investigated the absorbance changes in an aqueous 4-NP solution as a function of time. The absorbance of the solution at 400 nm in the presence of the PNIPAAm–AuNP hybrid microgel at 25 °C decreased, whereas that at 300 nm increased, as shown in Figure S9A. Importantly, the absorbance at 400 nm in the presence of the hybrid microgel with a small AuNP size (13.0 nm) decreased more rapidly than that with a large AuNP size (35.9 nm). This implies that the catalytic activity of the hybrid microgel with a small AuNP size is higher than that with a larger AuNP size. This is attributed to the fact that the increased surface area allows for a more efficient adsorption of 4-NP onto the AuNP surface, and that AuNPs with a smaller size have more active sites because their reduced dimensions correspond to lower coordination numbers. In contrast, the UV–Vis spectra of the aqueous 4-NP solution with the hybrid microgels at 40 °C did not change (Figure S9B), indicating that no reduction reaction occurred at 40 °C.

**Figure 5:**
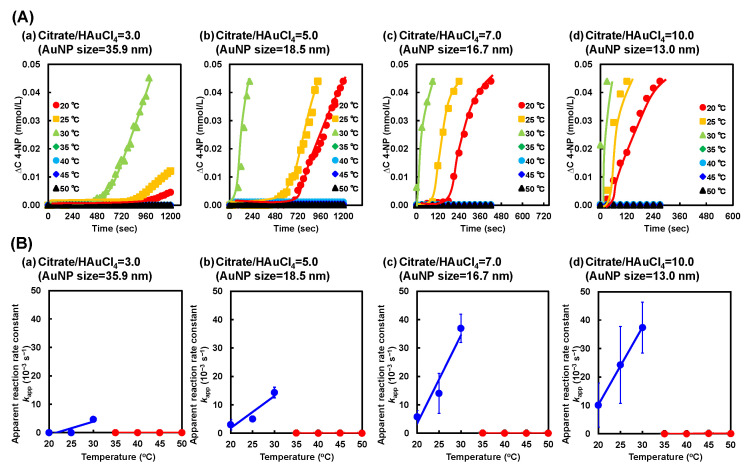
(**A**) Changes in 4-NP concentration during the reduction reaction of 4-NP to 4-AP using the PNIPAAm–AuNP hybrid microgel with various AuNP sizes at various temperatures. (**B**) Effect of temperature on the apparent reaction rate constant (*k*_app_) of reduction using the hybrid microgel with various AuNP sizes. AuNP monomers prepared with various citrate/HAuCl_4_ ratios (a–d) and AuNP sizes were used to prepare the hybrid microgels.

**Figure 6:**
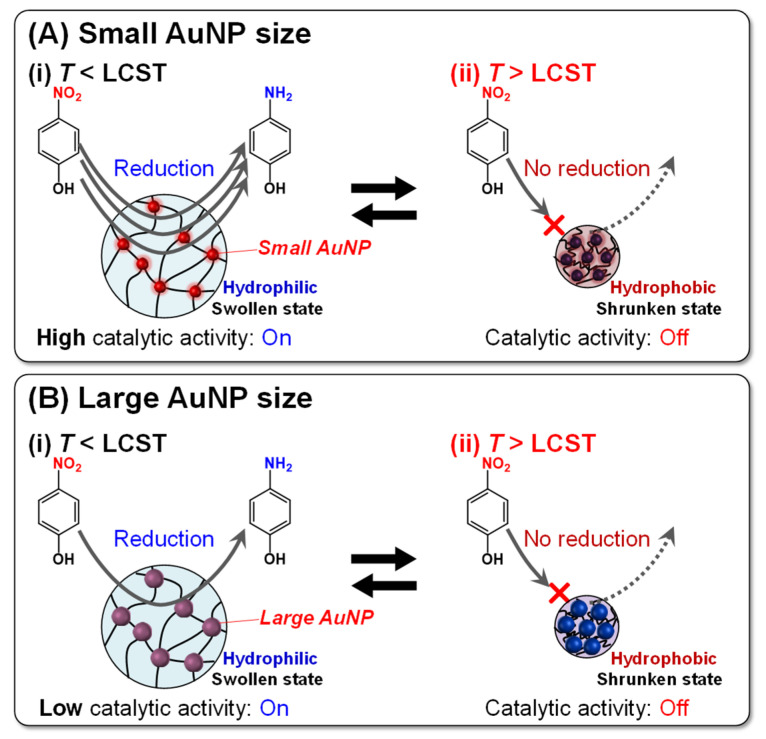
Reversible on–off regulation of catalytic activity of the PNIPAAm–AuNP hybrid microgels with small and large AuNP sizes in the reduction of 4-NP to 4-AP upon changes in temperature.

Next, we investigated the reaction kinetics of the reduction in 4-NP using hybrid microgels to clarify the temperature dependence of their catalytic activities. In general, the reaction kinetics for the reduction of 4-NP to 4-AP using catalysis can be described by a pseudo-first-order kinetic equation (ln(*C*_t_/*C*_0_) = ln(*A*_t_/*A*_0_) = −*kt*) [36]. In the reduction in 4-NP using hybrid microgels, the catalytic activity of microgels should be evaluated using a kinetic model in which the catalytic reaction is combined with the diffusion of 4-NP within the microgel networks. However, a quantitative evaluation of the diffusion of 4-NP within microgel networks is difficult because of the microsized and inhomogeneous networks. As a result, we evaluated the catalytic activity of the PNIPAAm–AuNP hybrid microgels by the “apparent” reaction rate including the contribution of 4-NP diffusion. The apparent reaction rate constant (*k*_app_) of the hybrid microgels was determined by monitoring the change in absorbance at 400 nm over time. Figure 5 shows the absorbance change in the aqueous 4-NP solution at 400 nm and the *k*_app_ of the hybrid microgels with different AuNP sizes during the reduction reaction at various temperatures. As described in our previous paper, induction times were observed in the reduction using hybrid microgels. As shown in Figure 5A, while the absorbance change in the hybrid microgel with small AuNP sizes exhibited little induction time, that with large AuNP sizes showed a long induction time. The hybrid microgel with large AuNP sizes exhibited lower catalytic activity, resulting in a longer induction time. On the other hand, as the hybrid microgel with small AuNP sizes had a larger surface area and higher catalytic activity, the 4-NPs had more opportunities to come into contact with the surface, which may have shortened the induction time. In this study, we determined the *k*_app_ of the reduction in 4-NP using the hybrid microgels from the initial slope of the time dependence of the change in absorbance at 400 nm after the induction time. The *k*_app_ of the reduction using the hybrid microgels increased with increasing temperature up to 30 °C, but became zero at temperatures higher than 30 °C (Figure 5B). As only PNIPAAm without AuNPs showed no catalytic activity in the reduction, the catalytic activity of the hybrid microgels was based on AuNPs within the microsized networks (Figure S10). In the reduction using hybrid microgels, the increase in *k*_app_ up to 30 °C is based on the well-known temperature dependence of the chemical reaction. Importantly, the *k*_app_ of the hybrid microgels with smaller AuNP sizes was greater than that with larger AuNP sizes at constant temperatures lower than the LCST of PNIPAAm. For example, the *k*_app_ for the reduction using the hybrid microgels with an AuNP size of 13.0, 16.7, 18.5, and 35.9 nm at 25 °C were approximately 24.2 × 10^−3^, 14.1 × 10^−3^, 5.0 × 10^−3^, and 1.3 × 10^−3^ s^−1^, respectively. Furthermore, at temperatures lower than 35 °C, the slope of the relationship between the temperature and *k*_app_ of the hybrid microgels became small with increasing the AuNP size, meaning that the *k*_app_ of the hybrid microgels with smaller AuNP sizes more strongly depended on temperature than that with larger AuNP sizes. These results indicate that the hybrid microgels with smaller AuNP sizes have higher catalytic activity than those with larger AuNP sizes. AuNPs with a smaller size have a greater total surface area than those with a larger size. Therefore, the higher catalytic activity of the hybrid microgels with smaller AuNP sizes is attributed to the fact that the increased surface area allows for a more efficient adsorption of 4-NP onto the AuNP surface and that AuNPs with a smaller size have more active sites because their reduced dimensions correspond to lower coordination numbers [59,62,63]. The catalytic activity of the hybrid microgels with a small AuNP size at temperatures lower than 35 °C was relatively high compared to the AuNP hybrid assemblies reported previously [55]. This might be attributed to the fact that the hybrid microgels have highly swollen networks and that 4-NP can easily approach the AuNP surface owing to their high permeability (Figure 6). More importantly, the *k*_app_ of the reduction using the hybrid microgels became zero at temperatures higher than 30 °C, unlike the general temperature dependence of chemical reactions. The PNIPAAm networks of the hybrid microgels shrank above the LCST of PNIPAAm because the PNIPAAm chains became more hydrophobic. The shrunken networks of the hybrid microgels prevent 4-NP molecules from approaching the AuNP surface [29,30,47,55]. As a result, the hybrid microgels with small AuNP sizes exhibited high catalytic activity and no catalytic activity below and above the LCST of PNIPAAm, respectively.

**Figure 7:**
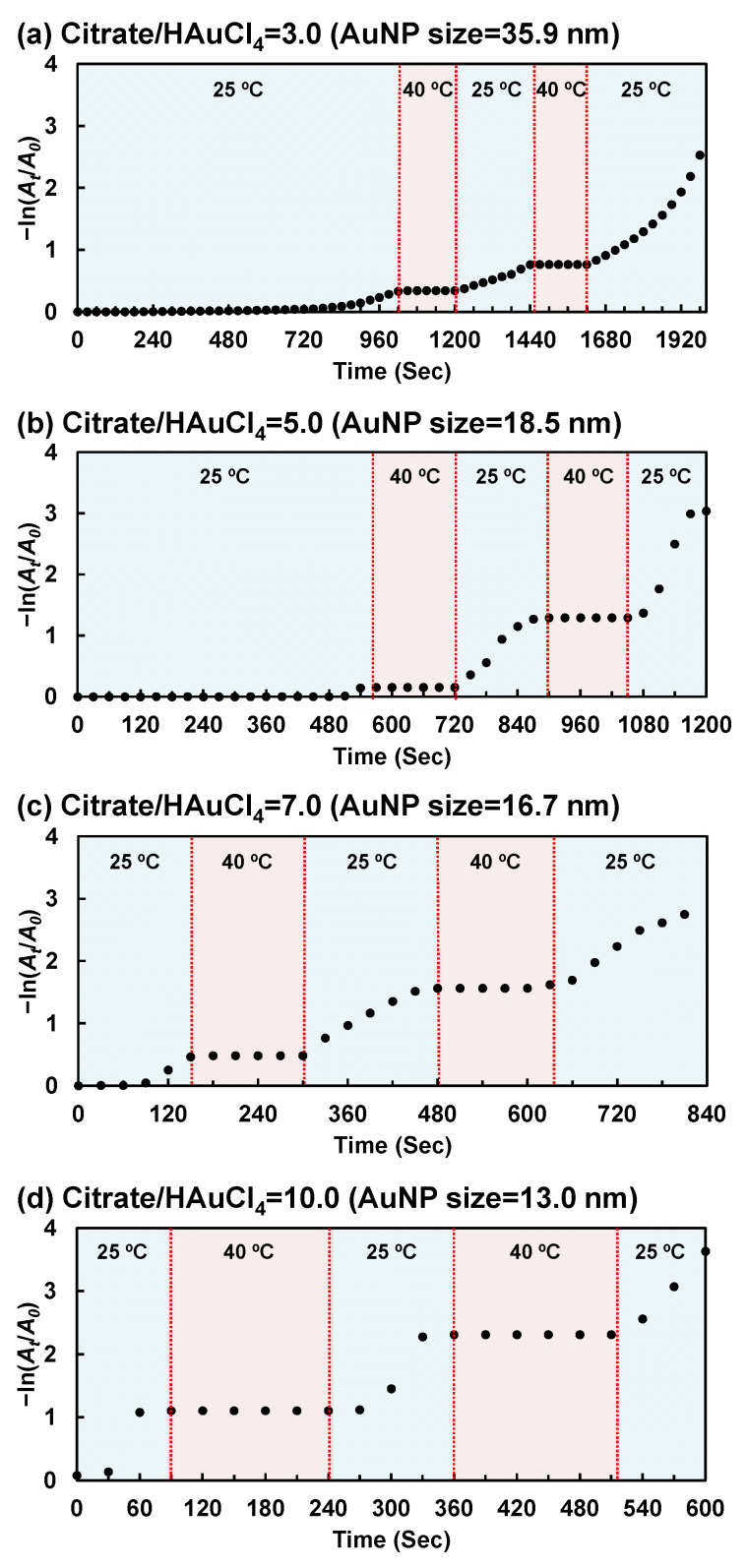
Changes in the 4-NP concentration during the reduction in 4-NP using the PNIPAAm–AuNP hybrid microgels upon stepwise changes in temperature between 25 and 40 °C. AuNP monomers prepared with various citrate/HAuCl_4_ ratios (**a**–**d**) and various AuNP sizes were used to prepare the hybrid microgels.

Next, we investigated the effect of AuNP size on the reversible temperature-responsive on–off regulation of the reduction reaction using the hybrid microgels. The reduction in 4-NP using the hybrid microgels with an AuNP size of 13.0, 16.7, 18.5, and 35.9 nm was monitored upon repeated changes between 25 °C and 40 °C (Figure 7). The hybrid microgels with different AuNP sizes catalyzed the reduction at 25 °C but not at 40 °C. Notably, the smaller the AuNP size of the hybrid microgel, the higher the catalytic activity. Importantly, the hybrid microgels exhibited a reversible on–off regulation of the reaction upon repeated changes in temperature. As a result, the temperature-responsive catalytic activity of the hybrid microgels can be improved by optimizing the AuNP size of the AuNP monomers. Even though the responsive catalytic activity of hybrid microgels still requires further research using molecular dynamics simulations and advanced spectroscopic techniques, they are likely to become important smart catalysts in the future.

## 3. Conclusions

The size of the AuNPs is a critical factor influencing the catalytic activity in the reduction of 4-NP to 4-AP. In this study, we designed AuNP monomers with different AuNP sizes and prepared PNIPAAm–AuNP hybrid microgels with different catalytic activities by the copolymerization of AuNP monomers and NIPAAm. AuNP monomers stably dispersed in water were designed by introducing acryloyl and carboxy groups onto the surface of citrate–AuNPs, whose size changed with the citrate/HAuCl_4_ ratio. The resulting AuNP monomers with different AuNP sizes were copolymerized with NIPAAm, followed by the formation of the hybrid microgels. Turbidity measurements revealed that an aqueous dispersion of the hybrid microgels changed from transparent to turbid at 34 °C with increasing temperature, and their transition temperature did not change at all with the AuNP size. The effect of the AuNP size on the catalytic activity of the hybrid microgels was investigated by monitoring the reduction in 4-NP as a function of temperature. The hybrid microgels exhibited catalytic activity in the reduction reaction below 34 °C, but had no activity above 34 °C. Importantly, below 34 °C, the catalytic activity of the hybrid microgels with smaller AuNP sizes was greater than that with larger AuNP sizes. In addition, the reversible on–off regulation of the reduction in 4-NP was achieved using the hybrid microgels with different AuNP sizes. This indicates that the reversibly temperature-responsive catalytic activity of the hybrid microgels can be enhanced using AuNP monomers with small AuNP sizes. Although further precise designs and structural optimizations of the AuNP monomers and hybrid microgels are required to enhance their catalytic activity and expand their practical applications, they are likely to have high potential as reversibly switchable catalysts.

## 4. Materials and Methods

### 4.1. Chemicals

Tetrachloroauric (III) acid trihydrate (HAuCl_4_ · 3H_2_O), sodium citrate hydrate, methanol (MeOH), iodine (I_2_), acryloyl chloride, triethylamine (TEA), dichloromethane (DCM), hydrochloric acid (HCl), NIPAAm, tetramethyl ethylenediamine (TEMED), ammonium persulfate (APS), 4-nitrophenol (4-NP), sodium borohydride (NaBH_4_), chloroform-d (CDCl_3_) 99.7% containing 0.05 vol% tetramethylsilane (TMS), and deuterium oxide (D_2_O) were purchased from Wako Pure Chemical Industries (Wako, Japan). In addition, 11-Mercapto-1-undecanol (MUD) and 16-mercaptohexadecanoic acid (MHDA) were purchased from Sigma-Aldrich (St. Louis, MO, USA). Silica gel and sea sand (425–850 μm) were purchased from Merck and Wako Pure Chemical Industries, respectively. All aqueous solutions were prepared using ultrapure water (18.2 MΩ/cm) produced from a Milli-Q water system. Other analytical-grade solvents and reagents were obtained from commercial sources and were used as received.

### 4.2. Preparation of AuNP Monomer and PNIPAAm–AuNP Hybrid Microgels

First, citrate–AuNPs of various sizes were prepared by the Turkevich method [74], as shown in Scheme S1. Then, 11, 11′-dithiodiundecyl dipropionate (DUDP) for introducing acryloyl groups onto the AuNP surface was synthesized by the method reported in our previous paper (Scheme S3) [55]. After aqueous solutions of sodium citrate hydrate with different concentrations (0.5, 0.6, 0.8, 1.3, 1.7, and 2.5 mmol/L) were prepared and refluxed at 120 °C for 30 min, a solution of HAuCl_4_·3H_2_O was added to attain the mixtures with a total Au ion concentration of 0.25 mmol/L and different molar ratios of sodium citrate hydrate to HAuCl_4_ (citrate/HAuCl_4_ ratios), followed by further refluxing for 15 min. Aqueous dispersions of the resulting citrate–AuNPs were subsequently purified by centrifugation at 9600 rpm at 20 °C for 20 min, and the supernatant was removed. After purification thrice using the same procedure, the resulting precipitated AuNP colloids were redispersed in ultrapure water to prepare an aqueous citrate–AuNP dispersion. The concentration of the citrate–AuNP in the aqueous dispersion, determined from the weight of the dispersion before and after the freeze-drying, was 0.35 mg/mL.

Acryloyl and carboxy groups were attached to the resulting citrate–AuNPs to prepare AuNP monomers with various AuNP sizes, as illustrated in Scheme S2 [55]. An aqueous citrate–AuNP dispersion (10 mL, 0.35 mg/mL) was added to a mixture containing DUDP/EtOH and MHDA/EtOH to prepare a 20 mL mixture with 0.25 mmol/L DUDP and 2.25 mmol/L MHDA. The mixture was stirred for 24 h to yield AuNP monomers of various sizes. The resulting AuNP monomers were purified using a previously reported procedure [55]. Aqueous dispersions of AuNP monomers with various AuNP sizes were prepared with 0.95 mg/mL.

PNIPAAm–AuNP hybrid microgels were prepared by the copolymerization of AuNP monomers with different AuNP sizes and NIPAAm (Scheme S4) using a previously reported method [55]. The concentration of the hybrid microgel in the aqueous dispersion, determined from the weight of the dispersion before and after freeze-drying, was 31.0 mg/mL.

### 4.3. Characterization of AuNP Monomers and PNIPAAm–AuNP Hybrid Microgels

The dispersibility of citrate–AuNPs, AuNP monomers, and PNIPAAm–AuNP hybrid microgels in water was evaluated by measuring the UV–Vis spectra of the dispersions using a UV-2550 spectrophotometer (Shimadzu Co., Kyoto, Japan). The diameters and polydispersity index (PDI) of the citrate–AuNPs (0.35 mg/mL), AuNP monomers (0.95 mg/mL), and hybrid microgels (0.31 mg/mL) were measured by dynamic light scattering (DLS) using an ELSZ-1000 spectrometer (Otsuka Electronics Co., Ltd., Osaka, Japan). Vertically polarized light at 633.8 nm from a He–Ne-ion laser was used as the incident beam, and all measurements were performed at 25 ± 0.3 °C. From the correlation function of light scattering in DLS measurements, the diameter and PDI were obtained by the cumulant method, and the size distribution was determined via Marquardt analysis.

The morphology of the citrate–AuNPs and hybrid microgels was observed by TEM using a JEM-1400F (JEOL Ltd., Tokyo, Japan) operated at an accelerating voltage of 100 kV. TEM samples were prepared by dropping an aqueous dispersion of citrate–AuNPs and hybrid microgels onto a carbon film-coated copper grid. The samples were dried prior to the experiment.

After freeze-drying, the hybrid microgels were characterized using the KBr method with FT-IR spectroscopy (Spectrum 100, PerkinElmer, Inc., Waltham, MA, USA). All the spectra represent an average of 32 scans taken in the wavenumber range of 450–4000 cm^−^^1^. The surface characterization of citrate–AuNPs and hybrid microgels was performed by X-ray photoelectron spectroscopy (XPS) using an ESCA-3400 (Shimadzu Co., Kyoto, Japan). An Mg Ka X-ray source was used at a power of 200 W (20 mA × 10 kV), and the pass energy was set at 75 eV. The pressure in the analysis chamber was ca. 4.0 × 10^−^^7^ Pa. Charge correction in the binding energy scale was performed by setting the –CH_2_– peak in the carbon spectra to 285.0 eV. The surface atomic ratios were determined from the integrated peak areas of C_1s_, N_1s_, O_1s_, S_2p_, and Au_4f_ and their respective experimental sensitivity factors. The fractional concentration of a particular element A, %*A*, was computed using Equation (1):

(1) $$\%A=\frac{I_{A}/s_{A}}{\sum \left(I_{n}/s_{n}\right)}$$

where *I_A_* and *s_n_* are the integrated peak areas and sensitivity factors, respectively.

The temperature-responsiveness of the hybrid microgels was evaluated by measuring the transmittance of the aqueous dispersions at 650 nm using a UV-2550 UV–Vis spectrophotometer (Shimadzu Co., Kyoto, Japan) when the dispersion was heated at a constant rate of 1 °C min^−1^. All tests were performed in triplicate.

### 4.4. Evaluation of Catalytic Activity of PNIPAAm–AuNP Hybrid Microgels

The catalytic activities of the PNIPAAm–AuNP hybrid microgels at various temperatures were evaluated by monitoring the reduction of 4-NP to 4-AP. After 1 mL of 10 mg/mL NaBH_4_ solution was added to 2 mL of 6.6 × 10^−5^ mol/L 4-NP solution in a quartz cell, 35 μL of an aqueous dispersion of AuNP monomers (0.95 mg/mL) or 100 μL of hybrid microgels (31.0 mg/mL) was added to the mixture. The concentrations of 4-NP and 4-AP were obtained from the absorbance at the maximum absorption wavelengths of 400 and 300 nm, respectively, using a UV-2550 UV–Vis spectrophotometer. In the reduction reaction of 4-NP, the ratio of absorbance (*A_t_*/*A*_0_) at time t corresponds directly to the ratio of the 4-NP concentration (*C_t_*/*C*_0_). Thus, the kinetic equation for reduction is expressed as Equation (2) [36]:

(2) $$r=ln\frac{C_{t}}{C_{0}}=ln\frac{A_{t}}{A_{0}}{=-k}_{\mathrm{app}}t\;$$

The apparent reaction rate constant, *k*_app_ (s^−^^1^), was obtained from the initial slope of *A_t_/A*_0_ as a function of time. All tests were carried out in triplicate.

## Data Availability

The data presented in this study are openly available in the article.

## Supplementary Materials

The following supporting information can be downloaded at: https://www.mdpi.com/article/10.3390/gels10060357/s1, Scheme S1: Preparation of citrate–AuNPs; Scheme S2: Preparation of AuNP monomer; Scheme S3: Synthesis of DUDP; Scheme S4: Preparation of PNIPAAm–AuNPs hybrid microgels; Figure S1: Photographs and UV–Vis spectra of aqueous dispersions containing citrate–AuNPs; Figure S2: Size distribution of citrate–AuNPs; Figure S3: ^1^H-NMR spectra of MUD, DUD, and DUDP; Figure S4: FT-IR spectra of MUD, DUD, and DUDP; Figure S5: FT-IR spectra of MHDA, DUDP, PNIPAAm, and PNIPAAm–AuNP hybrid; Figure S6: Hydrodynamic size and size distribution of the PNIPAAm–AuNP hybrid microgels; Figure S7: Photographs of aqueous dispersions of the PNIPAAm–AuNP hybrid microgels; Figure S8: Photographs of an aqueous 4-NP solution before and after the addition of the PNIPAAm–AuNP hybrid microgels; Figure S9: Absorbance changes during the reduction reaction of 4-NP using PNIPAAm–AuNP hybrid microgels; Figure S10: Absorbance changes during the reduction reaction of 4-NP in the presence of PNIPAAm; Table S1: Surface atomic ratios of the citrate–AuNP and PNIPAAm–AuNP hybrid microgel.
