# Supplementary material for: Effect of Gold Nanoparticle Size on Regulated Catalytic Activity of Temperature-Responsive Polymer−Gold Nanoparticle Hybrid Microgels
Source: Gels. 2024 May 22;10(6):357. doi: 10.3390/gels10060357 (PMC11202582; doi:10.3390/gels10060357)
Supplement: Supplementary file 1 [file gels-10-00357-s001.zip › gels-3010128-supplementary.pdf]

Supporting Information

Effect of Gold Nanoparticle Size on Regulated Catalytic Activity of Temperature-Responsive Polymer-Gold Nanoparticle Hybrid Microgels

Palida Pongsanon[1], *Akifumi Kawamura*[1,2], *Hideya Kawasaki*[1,2], *and Takashi Miyata*[1, 2,*]

[1] Department of Chemistry and Materials Engineering, Kansai University, 3-3-35, Yamate-cho, Suita, Osaka 564-8680, Japan

[2] Organization for Research and Development of Innovative Science and Technology, Kansai University, 3-3-35, Yamate-cho, Suita, Osaka 564-8680, Japan

*Phone: (+81) 6 63 68 09 49. Fax: (+81) 6 63 30 37 70. Email: tmiyata@kansai-u.ac.jp.

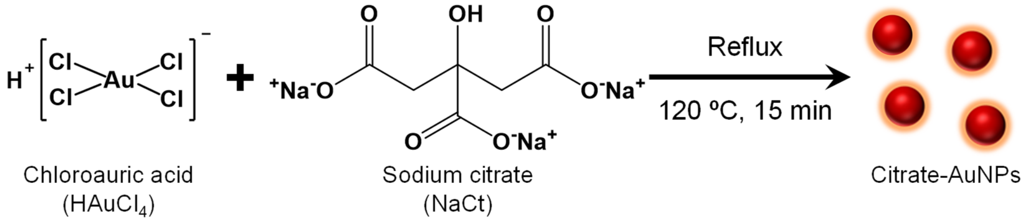

Scheme S1. Preparation of citrate-AuNPs.

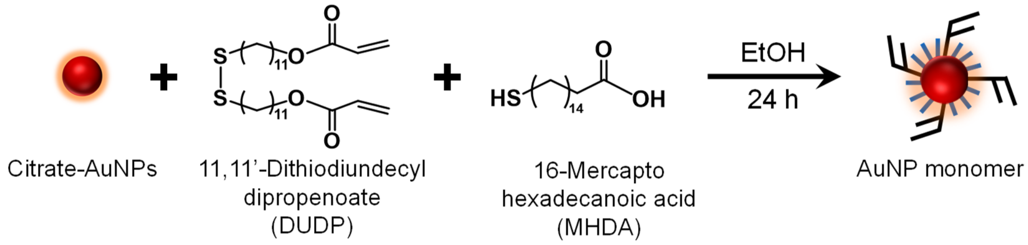

Scheme S2. Preparation of AuNP monomer.

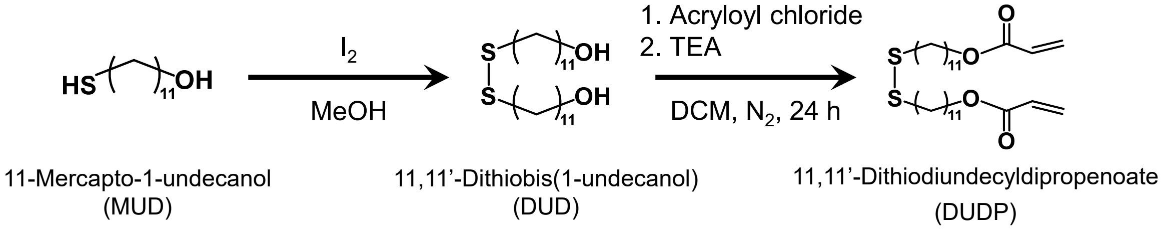

Scheme S3. Synthesis of 11,11'-dithiodiundecyl dipropenote (DUDP).

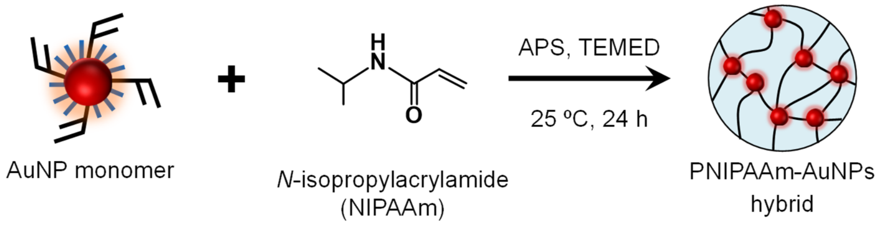

Scheme S4. Preparation of PNIPAAm-AuNPs hybrid microgels.

(A)

Citrate/$HAuCl_4$ ratio	2.0	2.5	3.0	5.0	7.0	10.0
Citrate (mmol/L)	0.5	0.6	0.8	1.3	1.7	2.5
$HAuCl_4$ (mmol/L)	0.25					
Citrate-AuNPs						

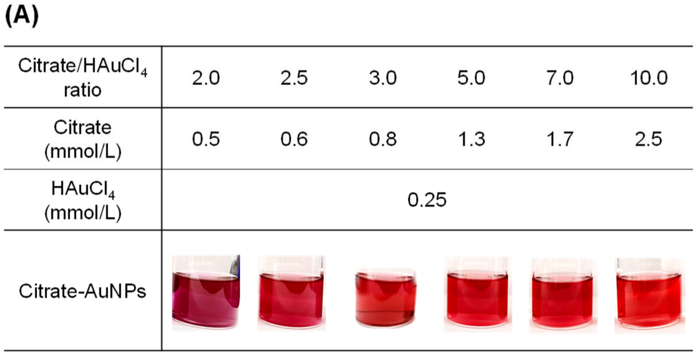

(B)

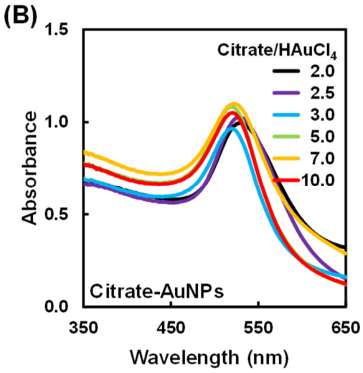

Figure S1. (A) Photographs and (B) UV-Vis spectra of aqueous dispersions containing citrate-AuNPs prepared with various citrate/$HAuCl_4$ ratios. The concentration of citrate-AuNPs was 0.35 mg/ml.

Citrate/$HAuCl_4$ ratio					
2.0	2.5	3.0	5.0	7.0	10.0
38.53 ± 5.84 nm	36.05 ± 10.17 nm	21.00 ±4.96 nm	19.98 ± 9.33 nm	14.84 ± 3.84 nm	12.95 ± 1.62 nm

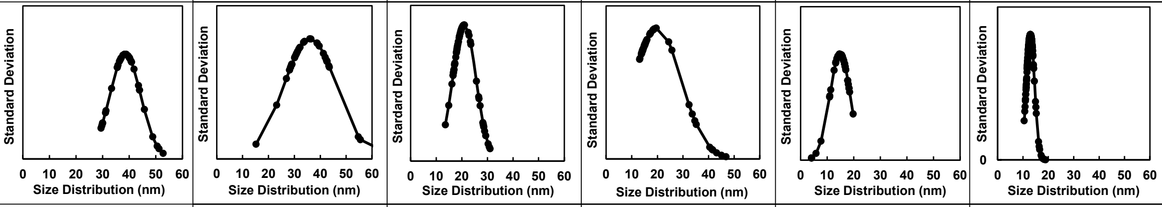

Figure S2. Size distributions of citrate-AuNPs determined by the analysis of the TEM images using a Java-based image processing program (ImageJ).

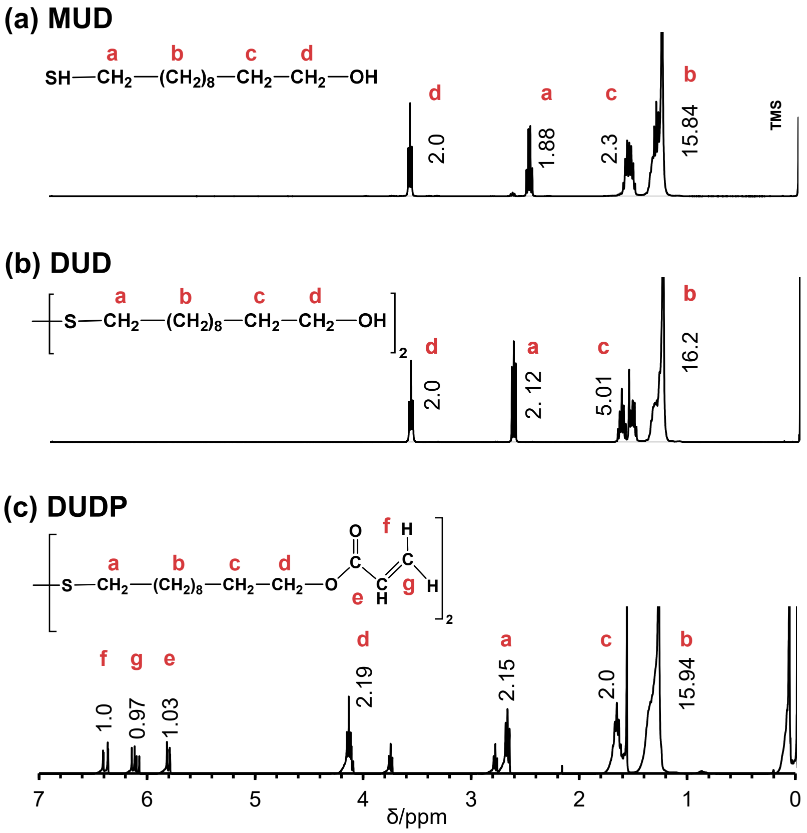

Figure S3. ^{1}H-NMR spectra of (a) MUD (400 MHz, $CDCl_3$, 16 times), (b) DUD (400 MHz, $CDCl_3$, 16 times), and (c) DUDP (400MHz, $CDCl_3$, 32 times).

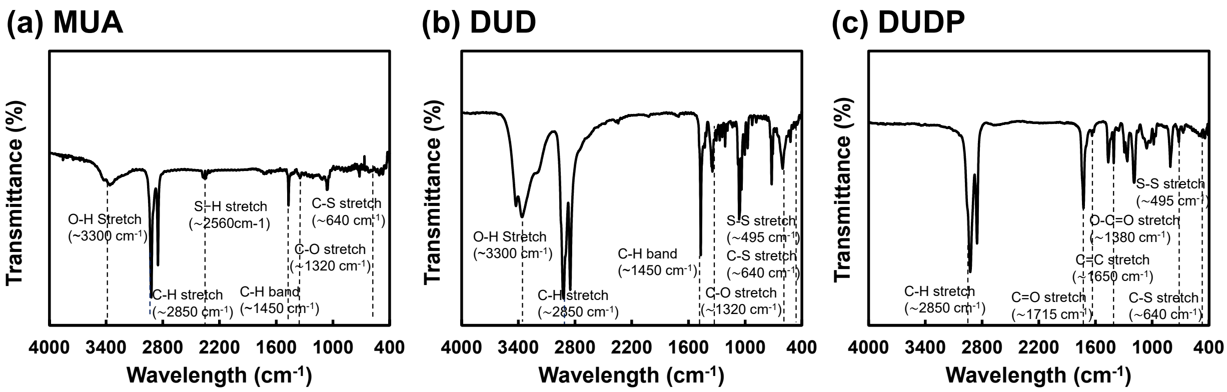

Figure S4. FT-IR spectra of (a) MUD, (b) DUD, and (c) DUDP.

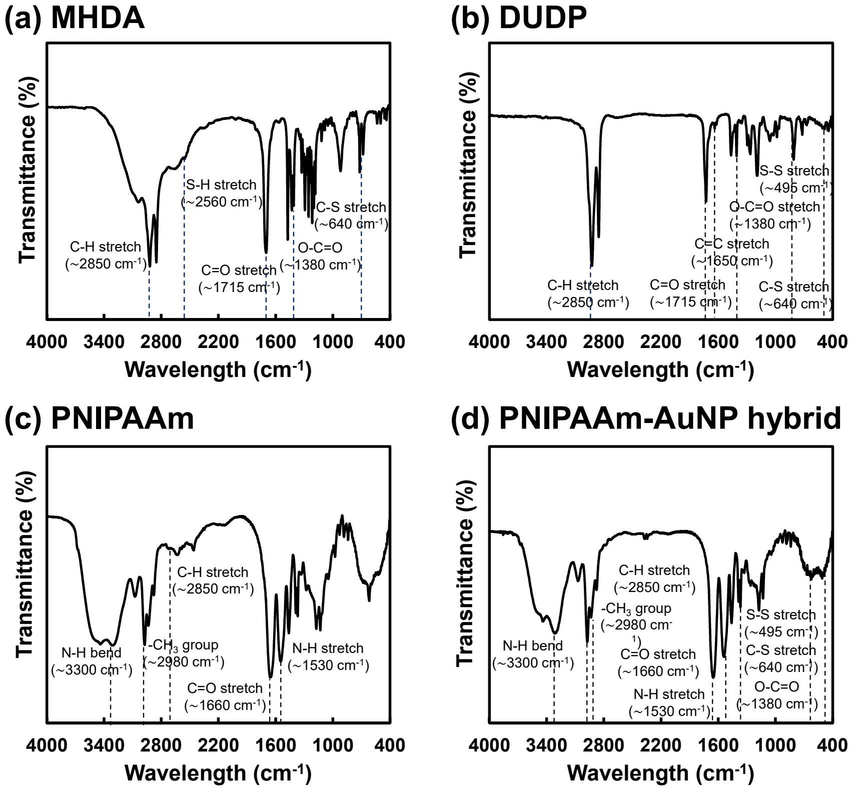

Figure S5. FT-IR spectra of (a) MHDA, (b) DUDP, (c) PNIPAAm, and (d) PNIPAAm-AuNP hybrid.

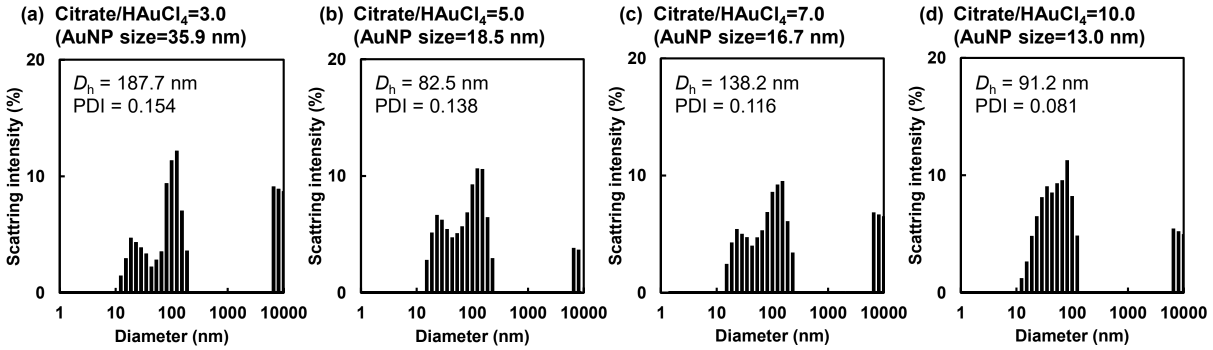

Figure S6. Hydrodynamic size (D_h) and size distribution of the PNIPAAm-AuNP hybrid microgels prepared using various AuNP monomers with a citrate/$HAuCl_4$ ratio of (a) 3.0, (b) 5.0, (c) 7.0, and (d) 10.0. The D_h and size distribution were determined by the DLS measurements with a PNIPAAm-AuNP hybrid microgel concentration of 1 mg/ml.

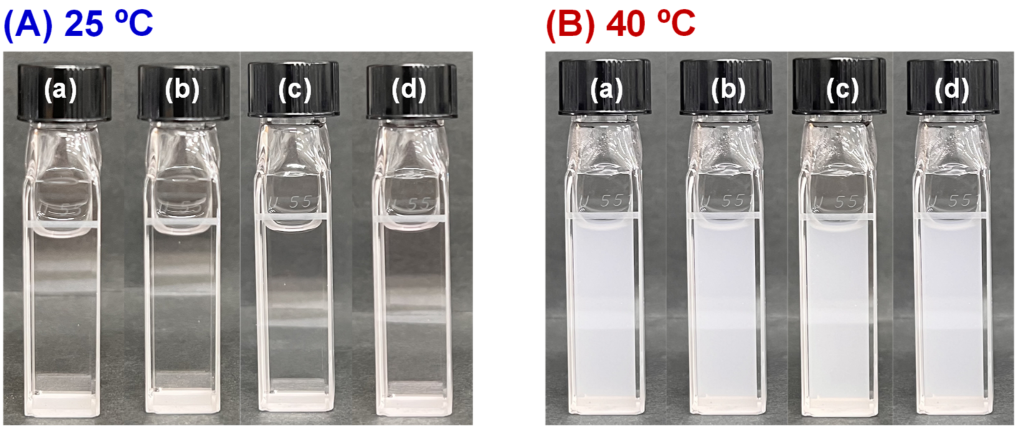

Figure S7. Photographs of aqueous dispersions of the PNIPAAm-AuNP hybrid microgels with a concentration of 1.0 mg/mL at (A) 25 and (B) 40 °C. The PNIPAAm-AuNP hybrid microgels were prepared using various AuNP monomers with a citrate/$HAuCl_4$ ratio of (a) 3.0, (b) 5.0, (c) 7.0, and (d) 10.0.

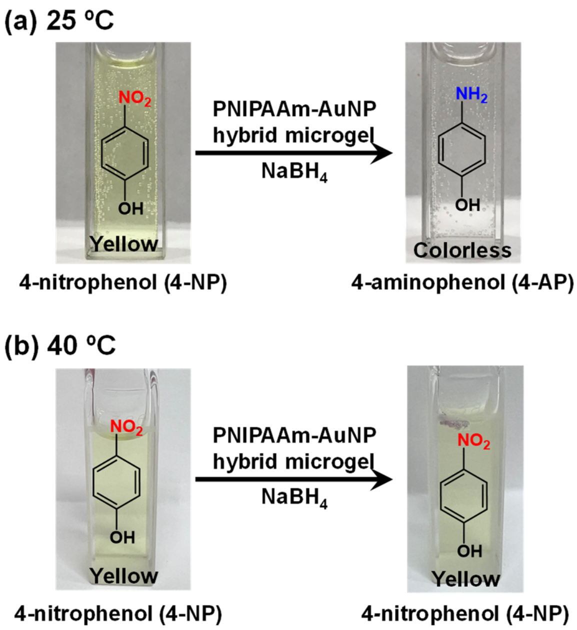

Figure S8. Representative photographs of an aqueous 4-NP solution with excess $NaBH_4$ before and after the addition of the PNIPAAm-AuNP hybrid microgels (1.0 mg/ml) at **(a)** 25 °C and **(b)** 40 °C. The PNIPAAm-AuNP hybrid microgels were prepared using the AuNP monomers with a citrate/$HAuCl_4$ ratio of 2.5.

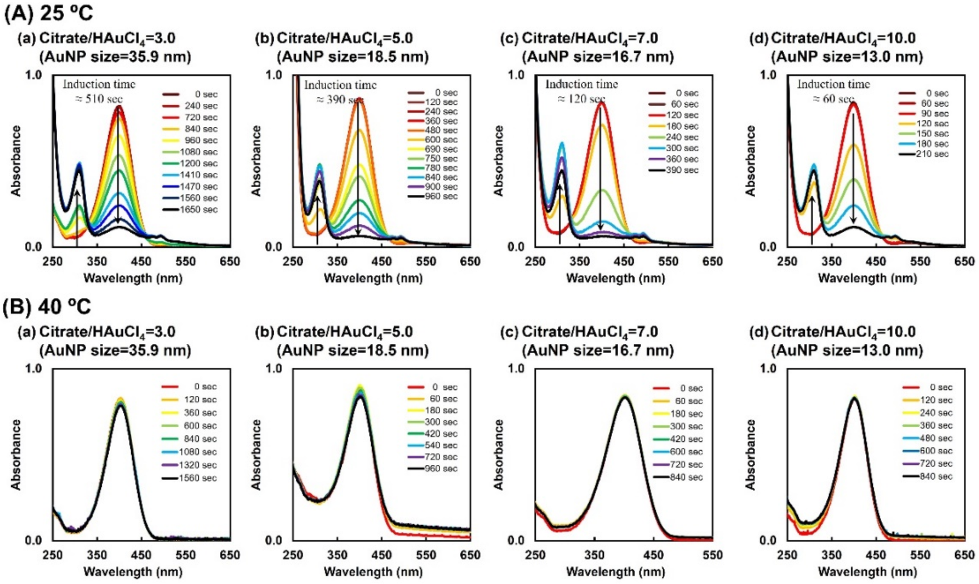

Figure S9. Absorbance changes during the reduction reaction of 4-NP to 4-AP using PNIPAAm-AuNP hybrid microgels at (A) 25 and (B) 40 °C. The AuNP monomers were prepared with DUDP and MHDA concentrations of 0.25 and 2.25 μmol/L, respectively, and the PNIPAAm-AuNP hybrid microgels were prepared using the AuNP monomer with various AuNP sizes (a-d).

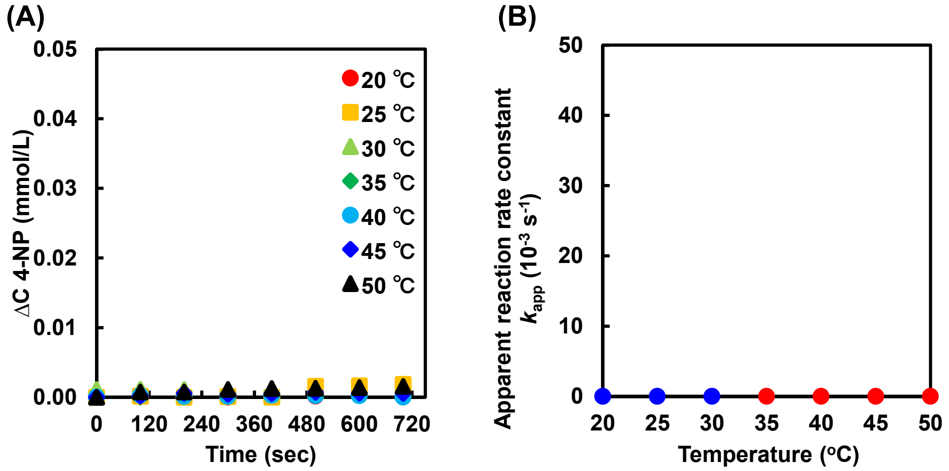

Figure S10. (A) Changes in 4-NP concentration during the reduction reaction of 4-NP to 4-AP using only PNIPAAm without AuNP at various temperatures. (B) Effect of temperature on the apparent reaction rate constant (kapp) of reduction using only PNIPAAm without AuNP.

Table S1. Surface atomic ratios of the citrate-AuNP and PNIPAAm-AuNP hybrid microgel by XPS measurements.

Particle	Citrate/$HAuCl_4$ ratio	C_{1s}	O_{1s}	N_{1s}	S_{2p}	Au_{4f}	C_{1s}/Au_{4f}	S_{2p}/Au_{4f}
**Citrate-AuNP**	3.0	66.4	22.1	-	-	11.5	5.8	-
	5.0	61.7	31.8	-	-	6.5	9.4	-
	7.0	57.2	29.9	-	-	12.9	4.4	-
	10.0	48.8	36.6	-	-	14.5	3.4	-
**PNIPAAm-AuNP hybrid microgel**	3.0	76.1	17.2	4.1	2.2	0.3	223.9	6.4
	5.0	73.1	21.9	2.7	1.6	0.7	105.9	2.3
	7.0	73.0	17.8	8.0	1.1	0.2	364.8	5.3
	10.0	73.5	20.8	3.2	2.1	0.4	170.9	5.0
